# Revealing the Correlation of OER with Magnetism: A New Descriptor of Curie/Neel Temperature for Magnetic Electrocatalysts

**DOI:** 10.1002/advs.202101000

**Published:** 2021-07-05

**Authors:** Xiaoning Li, Ying Bai, Zhenxiang Cheng

**Affiliations:** ^1^ International Joint Research Laboratory of New Energy Materials and Devices of Henan Province School of Physics and Electronics Henan University Kaifeng 475004 P. R. China; ^2^ Institute for Superconducting and Electronic Materials (ISEM) University of Wollongong Wollongong 2500 Australia

**Keywords:** Curie temperature, exchange interaction, hybridization, oxidization, oxygen evolution reaction

## Abstract

Developing accurate descriptors for oxygen evolution reaction (OER) is of great significance yet challenging, which roots in and also boosts the understanding of its intrinsic mechanisms. Despite various descriptors are reported, it still has limitations in the facile prediction, given that complicated analytical techniques as well as time‐consuming modeling and calculations are indispensable. In the present work, strong correlation of magnetic property with OER performance is revealed by in‐depth investigations on the crystal and electronic structures. A facile descriptor of Curie/Neel temperature (*T*_C/N_) is developed for La_2−_ *_x_*Sr*_x_*Co_2_O_6−_ *_*δ*_* perovskite oxides, based on the inference that both magnetism and OER are rooted in the electron exchange interaction. Specifically, both the *T*_C/N_ and OER activity are proportional to the degree of p‐d orbital hybridization, which increases with enlarged bond angle of Co─O─Co and/or increased oxidation of Co. This finding reveals that *T*_C/N_ from magnetic characterizations is an effective descriptor in designing novel OER electrocatalysts, and interdisciplinary researches are advantageous for revealing the controversial mechanisms of OER process.

## Graphical abstract

By comprehensive characterizations and analysis on crystal and electronic structures of La_2−_ *_x_*Sr*_x_*Co_2_O_6−_ *_*δ*_* perovskite oxides, the underlying physics for the relevance of magnetism with oxygen evolution reaction is revealed. A facile descriptor of Curie/Neel temperature is developed, which can easily be obtained from magnetization curves.

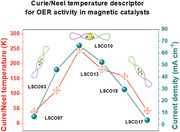

## 1 Introduction

Improving the efficiency of oxygen evolution reaction (OER) remains to be one of the highest priorities in the applications of energy conversion based on water splitting.^[^ ^1^, ^2^, ^3^ ^]^ The strong dependence of OER performances on the catalyst structures has been well recognized, together with numerous proposed descriptors.^[^ ^4^, ^5^, ^6^ ^]^ The significances of descriptors are instantiated in the more rational design of electrocatalysts by replacing traditional trial‐and‐error methods as well as in boosting the understanding of OER mechanisms.^[^ ^7^, ^8^ ^]^ Generally, the previously developed descriptors could be classified into two types. One type is associated with surface structures, such as the surface distortion,^[^ ^9^ ^]^ coordinatively unsaturated metal cation,^[^ ^10^ ^]^ coordination number,^[^ ^11^ ^]^ and so on. As OER occurs on the surface of electrocatalysts, the surface descriptors reflecting the local morphology and electronic environments are more accurate.^[^ ^12^ ^]^ However, due to the complexity of local surface, such as atom reconstructions, space charges, polarity, or segregation,^[^ ^13^ ^]^ a surface descriptor necessitates elaborate characterizations and in‐depth analysis by sophisticated surface‐sensitive techniques, which renders it more likes an evaluation tool rather than a material selection tool.

Another type is bulk descriptors such as the *e*_g_ filling, *N*–*V*, *p*‐band center, and charge‐transfer energy, which could be estimated from relative conventional measurements and calculations instead. For instance, *e*_g_ orbital filling with the optimum ≈1.2 can be estimated by bulk magnetic properties and X‐ray absorption analysis, based on the fact that *e*_g_ orbital is ready to form a strong spatial overlap with that of the O 2p from absorbates.^[^ ^14^, ^15^ ^]^ *N*–*V* descriptor reflects the net ability to donate electrons and the bond strength of B─O (B = transition metal in perovskites), corresponding to free energy difference (Δ*G*), wherein *N* is the number of unpaired electrons and *V* is the nominal valence charge of B cations on average.^[^ ^16^ ^]^ According to *N*–*V* descriptor, adsorption evolution mechanism dominates when Δ*G* > 0, while lattice oxygen mechanism is adopted when Δ*G* < 0.^[^ ^17^ ^]^ The O p‐band center descriptor predicts that an electrocatalyst will exhibit the highest activities when its O p‐band center is close to the Fermi level (*E*_F_), while the charge‐transfer energy is an effective descriptor for the transition of rate‐limiting steps, both of which are based on theoretical calculations.^[^ ^18^, ^19^ ^]^ Unfortunately, previously reported bulk descriptors are still lack of feasibility in facile prediction as they are requisite in complicated analytical techniques, time‐consuming modeling, and calculations. Hence, exploring new bulk descriptors with high efficiency, high accuracy, and easy handling is of great significance in practical application and theory research.

When exploring new descriptors for OER, we noticed that the magnetic property of a material is also very sensitive to the bulk/surface electronic structures, depending on the couplings of charge, spin, orbital, and lattice parameters, in a similar manner to OER.^[^ ^20^, ^21^, ^22^ ^]^ Interestingly, the association between OER and magnetism has been experimentally evidenced in a magnetic electrocatalyst NiZnFe_4_O*_x_*, with 40% improvement in the intrinsic OER activity when a moderate magnetic field (≤450 mT) was applied to the anode.^[^ ^23^ ^]^ At the same time, it is recognized that some of the descriptors including *e*_g_ electron number and average magnetic moment (Δ*μ*_ave_)^[^ ^24^ ^]^ are magnetic correlated factors in fact. Thus, other magnetic related descriptors that are easier in handling without complex calculation and hypothesis is anticipating. Herein, we focus on a La_2−_ *_x_*Sr*_x_*Co_2_O_6−_ *_*δ*_* material system with high OER activity and enriched magnetic properties,^[^ ^25^, ^26^, ^27^, ^28^ ^]^ to explore a more facile descriptor in effective predicting and selecting, as well as for better understanding in the OER mechanisms. Specifically, the evolution of crystal/electronic structures and variation of magnetic properties/OER performance are intensively characterized and analyzed. The results suggest that both OER activity and magnetism are based on the electron exchange interaction in the electrocatalysts,strongly correlated with the p‐d orbital hybridization. The parameters obtained from magnetic investigations are suggestive for the understanding of OER mechanisms. We proposed Curie/Neel temperature (*T*_C/N_) as an effective descriptor for OER electrocatalysts with ground magnetic states.

## 2 Results

### 2.1 Crystal Structure

**Figure 1:**
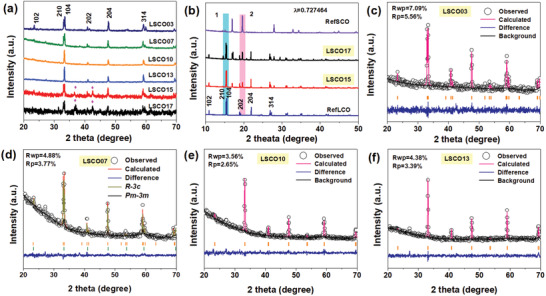
a) XRD patterns of as‐prepared samples. b) High‐resolution XRD patterns of LSCO15 and LSCO17 together with two reference samples. c–f) Refined XRD patterns of LSCO03, LSCO07, LSCO10, and LSCO13, respectively.

The as‐prepared La_2−_ *_x_*Sr*_x_*Co_2_O_6−_ *_*δ*_* powder samples with *x* valued at 0, 0.3, 0.7, 1.0, 1.3, 1.5, 1.7, and 2 are denoted as RefLCO, LSCO03, LSCO07, LSCO10, LSCO13, LSCO15, LSCO17, and RefSCO, respectively. From the X‐ray diffraction (XRD) patterns in **Figure** **1**a we can see that the LSCO03, LSCO07, LSCO10, and LSCO13 are of pure perovskite phases without detectable impurity. However, some tiny peaks are observed in the patterns of LSCO15 and LSCO17 samples (as marked in pink rhombus) which should be arose from heavy Sr substitution. To identify their possible phases, high‐resolution XRD patterns used the synchrotron radiation X‐ray source (*λ* = 0.727464 nm) of RefLCO, LSCO15, LSCO17, and RefSCO are measured and displayed in Figure 1b. By comparing their patterns, it is found that all the diffraction peaks from LSCO15 and LSCO17 can be well assigned to RefLCO and RefSCO. Herein, RefLCO is a pure perovskite phase, while RefSCO is an oxygen vacancy‐enriched perovskite phase.^[^ ^29^ ^]^ By calculating the intensity ratio of two peaks located at peak 1 that is identical to RefLCO phase and peak 2 that is identical to RefSCO phase, the content of oxygen vacancy‐enriched perovskite phase is roughly estimated to be about 25% and 31% in the LSCO15 and LSCO17, respectively ( = *I*_2_/(*I*_1_+*I*_2_)×100%).

For the other samples, their crystal structures can be well revealed by refining XRD patterns. As shown in Figure 1c,e,f, when refined based on the space group of *R‐3c* for LSCO03, and *Pm‐3m* for LSCO10 and LSCO13, the resultant *R*_wp_ and *R*_p_ values are smaller than 7%, confirming their single perovskite phase. According to the lattice parameters listed in Table S1 in the Supporting Information, accompanied with the conversion of space group, the bond angle of Co─O─Co and bond length of Co─O are generally enlarged with the increment of Sr. This may be derived from the larger ionic radius of Sr compared with that of La (radii in 1.18 vs 1.03, hexa‐coordination). In the case of LSCO07 sample, it is refined based on two perovskite phases with space group of *R‐3c* (77%) and *Pm‐3m* (23%) (Figure 1d). With medium amount of Sr, LSCO07 sustains a partial lattice transformation from *R‐3c* (in the case of LSCO 03) to *Pm‐3m* (in the case of LSCO 10). From above structure analysis it can be seen that all the as‐prepared samples are perovskite structures, despite some with single phase, and some with multiple phases.

### 2.2 Electronic Structure

**Figure 2:**
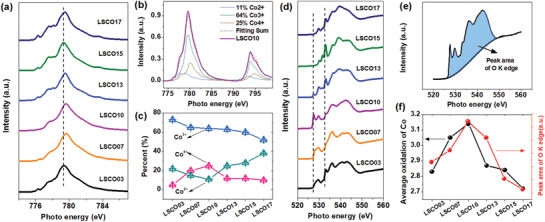
a) Co *L_3_*‐edge XANES spectra. b) The fitting of Co *L*‐edge XANES spectrum of LSCO10 based on 11% Co^2+^, 64% Co^3+^, and 25% Co^4+^. c) The evolution of Co^2+^, Co^3+^, and Co^4+^. d) O *K*‐edge XANES spectra. e) Demonstration of peak area of O *K*‐edge of LSCO10. f) The evolution of average oxidation of Co and peak area of O *K*‐edge.

**Figure****2**a exhibits the X‐ray absorption near edge structure (XANES) spectra of Co *L*_3_‐edge, collected by the total electron yield mode which is sensitive to the surface (≈5 nm). Although the Co *L*_3_‐edge spectra for all the samples display a similar shape, the main peak (marked as dash lines around 779.4 eV) is shifted gradually to the higher energy direction from LSCO03 to LSCO17, indicating the change of oxidation states. To quantitatively analyze this evolution process, QANT software developed by Soft X‐ray beamline of Australia Synchrotron (AS) was applied,^[^ ^30^ ^]^ together with the XANES spectra of three standard samples CoO (Co^2+^), Co_2_O_3_ (Co^3+^), and SrCoO_3_ (Sr^4+^) as the fitting components. As demonstrated in Figure 2b, the surface of LSCO10 is estimated to be involved of 11% Co^2+^, 64% Co^3+^, and 25% Co^4+^ (for the fitting results of other samples, see Figure S1, Supporting Information). Figure 2c depicts the variation tendency of the proportions of Co^2+^, Co^3+^, and Co^4+^ from LSCO03 to LSCO17. It is obvious that the percentage of Co^3+^ keeps decreasing over all range with the increase of Sr content. However, the percentage of Co^2+^ deceases at first from LSCO03 to LSCO07, and increases to LSCO17 afterward. Meanwhile Co^4+^ has the exactly opposite tendency to that of Co^2+^, increasing at first and decreasing afterward. This phenomenon can be well explained by the electroneutrality principle that the extra charges introduced by the substitution of Sr^2+^ have to be compensated by means of the generation of Co^4+^ and/or oxygen vacancies. When the amount of Co^4+^ reaches its limitation, massive oxygen vacancies emerge, transforming adjacent Co^4+^ into Co^2+^ as a consequence.^[^ ^31^ ^]^ In this case, maximum of Co^4+^ and minimum of Co^2+^ are achieved in the LSCO10 sample.

O *K*‐edge XANES is robust in characterizing the hybridization of unoccupied p‐d orbitals (conduction band). As shown in Figure 2d, the first peak (≈530 eV) corresponds to the excitation from O 1s states to unoccupied hybridized O 2p−Co 3d states. The second (≈535 eV) and third (≈543 eV) peaks are attributed to the excitations of O 1s to unoccupied O 2p−La 5d and O 2p−Co 4sp, respectively.^[^ ^26^ ^]^ Specifically, the peak ≈528 eV is associated with the unoccupied *t*_2g_ orbitals in the octahedral coordination. Its intensity increases at first and decreases afterward, similarly to the tendency of Co^4+^ as mentioned above. This can be interpreted by the fact that Co^4+^ has relatively more unoccupied *t*_2g_ orbitals compared with Co^3+^ and Co^2+^ if they are with the same spin states. The band at 533.2 eV could be attributed to the excitation from O 1s to the oxygen‐vacancy‐correlated unoccupied O 2p orbitals, the intensity of which decays at first from LSCO03 to LSCO10 and then all way up to LSCO15. This trend is in well agreement with the above result that where there is more Co^4+^, there are less oxygen vacancies. The slight decrease in LSCO17 is likely due to the mediation of oxygen intercalation from the absorbates with excess surface vacancies.^[^ ^31^ ^]^ Additionally, the hybridization of O 2p‐Co 3d could be roughly estimated by the integration of peak areas as demonstrated in Figure 2e, assuming that all the occupied and unoccupied states are of the same hybridization extent and the spectral intensity is linearly proportional to the total number of the unoccupied state. The resulted integration of peak area in O *K*‐edge and the average valence states of Co are concordant with each other (Figure 2f), which is in agreement with the previous report,^[^ ^32^ ^]^ achieving maximum in the LSCO10 sample. All these consistent results suggest that LSCO10 exhibits highest oxidized Co and strongest hybridization of unoccupied O 2p‐Co 3d states.

**Figure 3:**
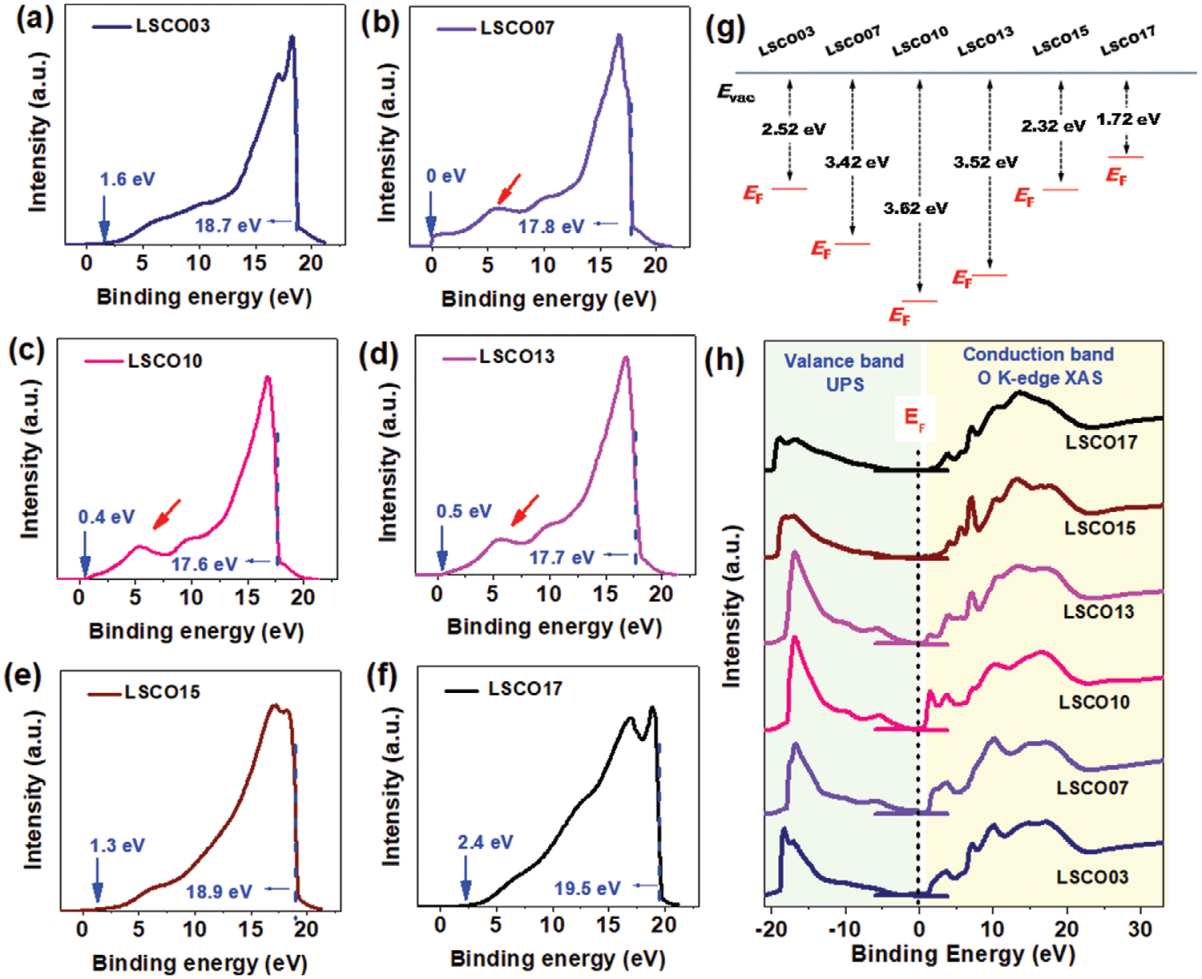
a–f) UPS spectra; g) partial band diagrams; h) the band structure near the *E*_F_ by combining the experimental UPS and O *K*‐edge XAS spectra.

Ultraviolet photoemission spectroscopy (UPS) is another widely used surface analysis technique, especially for the study of valence band density of states and the work function (WF), which can also provide information of the hybridization of occupied p‐d orbitals (valence band).^[^ ^33^ ^]^ The UPS spectra in **Figure** **3**a–f are calibrated by standard Ag substrate with a WF value of 4.26 eV. The feature of d orbitals with sharp peaks is obvious in the LSCO07, LSCO10, and LSCO13 (marked in red arrows) samples, indicating the strong O 2p‐Co 3d hybridization. The positions of valance band maximum or highest occupied molecular orbital relative to Fermi levels (*E*_F_) are marked in blue arrows, and the cut‐off edges (*E*_cut‐off_) are drawn in blue dashed lines at high binding energy range. According to their definitions, the position of *E*_F_ level is equal to the negative value of WF, which could be calculated based on the following Equation (1)^[^ ^34^ ^]^

(1) $$E_{F}=-\mathrm{WF}=-\left(hv\;-\;E_{\mathrm{cut}-\mathrm{off}}\right)$$

wherein *hv* is the incident photoelectron energy (21.22 eV). The WF is estimated to be 2.52, 3.42, 3.63, 3.52, 2.32, and 1.72 eV for the as‐prepared LSCO03, LSCO07, LSCO10, LSCO13, LSCO15, and LSCO17, respectively. As illustrated in Figure 3g, the *E*_F_ is lowered from LSCO03 to LSCO10 and elevated afterward, reaching the minimum in LSCO10. The results from UPS further confirm that LSCO10 has the strongest hybridization of occupied O 2p‐Co 3d states.

Besides, the band structure near the *E*_F_ can be roughly demonstrated by combining the experimental UPS and O *K*‐edge X‐ray absorption spectra (XAS) spectra, as the O *K*‐edge XAS spectrum is comparative to the empty states in the density functional theory calculated partial density of states.^[^ ^35^ ^]^ The original photon energy axis in the O *K*‐edge XAS spectra can be converted to the binding energy by subtracting of the onset position of O 1s core level spectrum (526.0 eV, see Figure S2, Supporting Information). As shown in Figure 3h, it is remarkable that the uppermost region in the valance band and the lowermost region in the conduction band near the *E*_F_ are both strongly hybridized O 2p−Co 3d band in nature for all the samples, suggesting a p−p type of charge fluctuations.^[^ ^36^ ^]^ In the strong electron‐correlated material systems, the on‐site Coulomb potential (*U*_dd_) localizes the d bands, leading to a d−d gap between occupied and empty ones.^[^ ^37^ ^]^ Due to the strong hybridization of p‐d orbits, the gap between the highest occupied states and the lowest unoccupied states is narrowed utmost in the LSCO10 sample.

### 2.3 Electrochemical Performances

**Figure 4:**
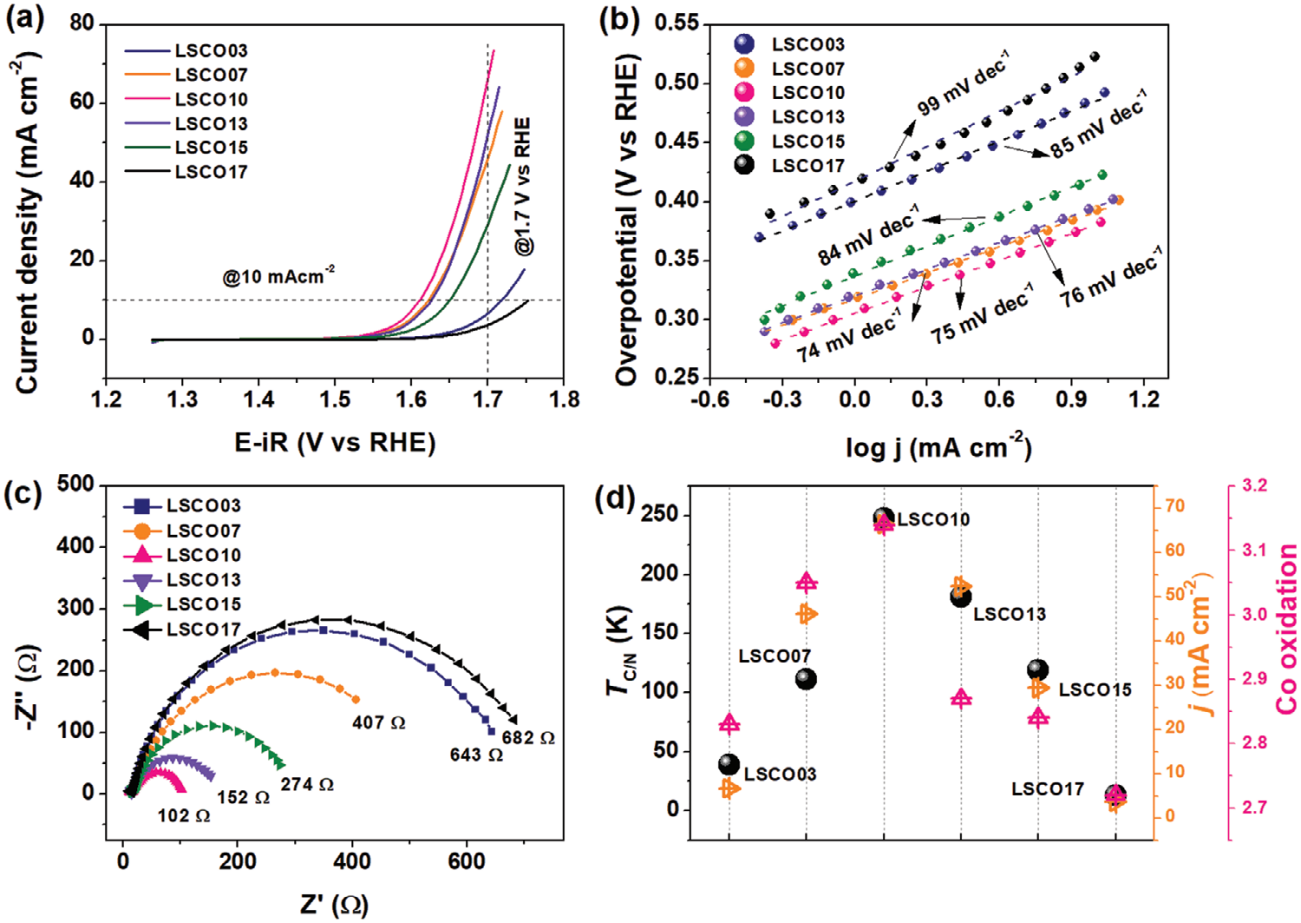
a) LSV curves with a scan rate of 5 mV s^–1^ in 1 m NaOH. b) Tafel plots. c) Nyquist plots at 1.6 V versus RHE. d) Evolution of electronic and OER evaluation parameters.

The OER performances are tested by linear sweep voltammetry (LSV) at first with a scan rate of 5 mV s^–1^ in the 1 m NaOH medium. As shown in **Figure** **4**a, there is no redox peak emerged before 1.5 V versus reversible hydrogen electrode (RHE). Therefore, the current collected beyond 1.5 V versus RHE could be unexceptionally assigned to the OER catalyzed by as‐prepared electrocatalysts. Normalized by the geometric area of GC electrode, the current density (*j*) at certain applied potential (1.7 V vs RHE, e.g.) is a basic parameter for reaction activity evaluation. As signified by the dash line positioned at 1.7 V versus RHE in Figure 4a, the best OER performance is achieved on the LSCO10 sample with the highest current density. Specifically, the *j* at 1.7 V versus RHE is about 7, 46, 66, 52, 29, and 4 mA cm^–2^ for LSCO03, LSCO07, LSCO10, LSCO13, LSCO15, and LSCO17 samples, respectively. The overpotential (*η*) at the current density *j* = 10 mA cm^–2^ is another practical evaluation parameter, which again confirms the best OER activity of LSCO10 under the smallest applied potential. Specifically, the values of *η* at 10 mA cm^–2^ are 488, 392, 381, 395, 418, and 523 mV for LSCO03, LSCO07, LSCO10, LSCO13, LSCO15, and LSCO17, respectively.

The Tafel plots are displayed in Figure 4b, with slopes of 85, 74, 75, 76, 84, and 99 mV dec^–1^ for LSCO03, LSCO07, LSCO10, LSCO13, LSCO15, and LSCO17, respectively. With reduced Tafel slopes, the OER kinetics for LSCO07, LSCO10, and LSCO13 should be much faster than the others, among which LSCO10 is fastest, consistent with the analysis of LSV. Nyquist plots in Figure 4c obtained at 1.6 V versus RHE can be resolved by several components. The polarization resistances (semicircle diameters) are fitted to be 643, 407, 102, 152, 274, and 682 Ω for LSCO03, LSCO07, LSCO10, LSCO13, LSCO15, and LSCO17, respectively. The greatly reduced resistance during polarization implies a lowest electron transfer barrier in the LSCO10 sample.

In search of facile OER activity descriptors from magnetism, we have fully characterized the magnetic property of these samples, intensive analysis and discussions are provided in the Supporting Information. For clarity, the current density (*j* at 1.7 V vs RHE) representative for OER activity, average oxidation state of Co representative for intrinsic electronic structures, and Curie/Neel temperature (*T*_C/N_) are compared. As shown in Figure 4d, it is observed that OER parameter are well accordant with electronic structure parameter, as well as the Curie/Neel temperature (*T*_C/N_). As we all know, Curie/Neel temperature (*T*_C/N_) can be easily obtained from the magnetization curves, as discussed in detail in the Supporting Information. Here, it is noteworthy to mention that the existence of multiple perovskite phases does not influence above observations. Generally, the differences between different perovskites lie in the Co─O bonding, which has been already reflected on the more intrinsic geometric and electronic evolutions, such as the oxidation state of Co and hybridization of 2p‐3d. The underlying intrinsic physics are discussed as follows to explain these observations.

## 3 Discussions

The consistency of the oxidation, hybridization, *T*_C/N_, and OER activity can be reckoned as follows. In the perovskite lattice, six O anions binding with one Co cation forms an octahedron (CoO_6_), meanwhile eight adjacent La/Sr cations build up a hexahedron unit cell frame. When the bond length of Co─O is larger than that of La/Sr─O, i.e., the octahedron is larger than hexahedron, CoO_6_ octahedrons will be distorted or even collapse. The radius of a Co cation is directly determined by its oxidation state, which is smaller for the higher oxidization. Thus, with the increment of Sr content from LSCO03 to LSCO17, the bond length of Co─O is supposed to be shortened as the oxidation increases. However, the average bond length of La/Sr─O is also elongated simultaneously, as the radius of Sr^2+^ is larger than that of La^3+^. In this case, the integrated/synergetic effect of enlarged hexahedron frame and shrinking octahedron leads to a variation of bond angle and space group as indicated by XRD results. The hybridization of 2p‐3d orbitals not only depends upon the Co─O bonding, but also reflects the state of Co─O bonding in turn. Due to unique orbital shape of p and d orbitals, 2p‐3d hybridization is stronger in the cases of the shorter bond length or larger bond angle. Thus, the evolution of crystal and electronic structures can be projected onto the orbital hybridization parameters.

In the meantime, the hybridization can be projected onto the magnetic parameters, and their correlation can be estimated according to Heisenberg model and theory (Equations (2)–(4))

(2) $$T_{C}=\frac{zA}{2k_{B}}$$

(3) $$A=\int \;\;\;\;\int \varphi_{a}^{\prime }\left(1\right)\varphi_{b}^{\prime }\left(2\right)\cdot V_{\mathrm{ab}}\cdot \varphi_{a}\left(2\right)\varphi_{b}\left(1\right)d\tau_{2}d\tau_{1}$$

(4) $$V_{\mathrm{ab}}=e^{2}\;\left(\frac{1}{r_{12}}-\frac{1}{r_{b1}}-\frac{1}{r_{a2}}\right)$$

Herein, *z* is the coordination number (*z* = 6), *A* is the exchange integral, *k*_B_ is the Boltzmann constant, *r*_12_ represents the distance of two electrons (1, 2) from two adjacent cations (a, b), *r*_b1_ represents the distance of electron (1) from cation (a) to the nucleus of cation (b), and *r*_a2_ represents the distance of one electron (2) from cation (b) to the nucleus of cation (a). Consequently, the Curie/Neel temperature (*T*_C/N_) is proportional to the exchange integral *A*, which is decided by the orbital hybridization. While the orbital hybridization is stronger when the nuclei are closer or when electrons are far away from nucleus and closer to each other. The above equations and analysis clearly elucidate the strong correlation of Curie/Neel temperature (*T*_C/N_) with the orbital hybridization.

For the OER, surface adsorption/desorption are deemed to be the most critical steps in determining the efficiency, which is actually defined as “exchange interaction” of electrons between oxygen species and the active sites at the interface. The orbital hybridization of 2p‐3d is supposed to be one of the main determining factors for the exchange interaction in the interface as disscussed above, wherein the 2p orbital is from oxygen species instead of electrocatalysts. For the “electron transportation” in the bulk crystal lattice afterward, it is also rooted in the exchange interaction thus the orbital hybridization of 2p‐3d, where the 2p orbital is from lattice oxygen of electrocatalysts. Thus, the strong hybridization triggers fast electron transfer at the interface and transport in the bulk during the whole OER process. Many well‐acknowledged influencing factors/descriptors for OER, such as higher oxidation and shorten bond length, may also be inevitably resulted in stronger hybridization, as the intrinsic parameters (charge, spin, orbital, and lattice) are strongly coupled.

Therefore, deriving from the analogous physical origins, that is the exchange interaction, the magnetic property investigations could be fully taken advantage to unveil the OER theory. Herein, the Curie/Neel temperature (*T*_C/N_) is proposed as an efficient and accurate OER activity descriptor in the materials with magnetic ground states (ferro/ferri/antiferro‐magnetism), which could be easily obtained without complicated calculation or other advanced characterization techniques.

## 4 Conclusions

With intensive investigations on the crystal structure by high‐resolution XRD, electronic structure by XAS/UPS, magnetic properties, and OER performance, consistent results are achieved on the magnetic electrocatalysts La_2−_ *_x_*Sr*_x_*Co_2_O_6−_ *_*δ*_*. When the bond angle is enlarged, the oxidation is increased, the hybridization of p‐d orbital is stronger. Both the magnetism and OER are found rooted in the electron exchange interaction, which is proportional to the orbital hybridization. The Curie/Neel temperature (*T*_C/N_), which could be easily obtained from magnetization curves, is established to be an effective descriptor of OER activity for magnetic materials. The findings in this work not only pave the way to build strong bonds between the magnetic property and OER mechanisms, but also shed light in screening novel OER catalyst for energy conversion in the water splitting field.

## 5 Experimental Section

### Sample Preparation

All the used chemicals including the La(NO_3_)_2_·6H_2_O (99.99%), Sr(NO_3_)_2_ (≥99%) Co(NO_3_)_2_·6H_2_O (≥98%), and Nafion 117 solution were purchased from Aldrich Ltd. Certain amount of raw materials were mixed according to the stoichiometric ratio of La_2−_ *_x_*Sr*_x_*Co_2_O_6−_ *_*δ*_* (*x* = 0, 0.3, 0.7, 1.0, 1.3, 1.5, 1.7, and 2), with a subsequent heat‐treatment at 973 K for 2 h. After cooled down to room temperature, the calcined powder was ground thoroughly and compressed into a disk under an axial pressure of 10 MPa. Then, the disk was sintered at 1233 K for 12 h and ground into a fine powder again when cooled down. The final La_2−_ *_x_*Sr*_x_*Co_2_O_6−_ *_*δ*_* powder samples with *x* valued at 0, 0.3, 0.7, 1.0, 1.3, 1.5, 1.7, and 2 were denoted as RefLCO, LSCO03, LSCO07, LSCO10, LSCO13, LSCO15, LSCO17, and RefSCO, respectively.

### Physical Characterizations

XRD patterns were recorded on a PANalytical Empyrean X‐ray diffractometer with Cu‐K*α* radiation. High‐resolution XRD was detected from powder diffraction beamline at the Australian Synchrotron (AS, Australia), part of ANSTO. Co *L*‐edge and O *K*‐edge soft XAS were collected from the soft X‐ray spectroscopy beamline at the Australian Synchrotron (AS, Australia), part of ANSTO. The measurements of X‐ray photoelectron spectroscopy and UPS were conducted on a NEXSA X‐ray photoelectron spectrometer (Thermo Scientific) on fresh surface of the samples after ion etching. For the UPS, He I (*hν* = 21.22 eV) photon line of a He discharge lamp was used and a bias voltage of −10 eV was applied. A clean Ag substrate was also tested for the calibration of the Fermi level. Magnetization–temperature curves in the zero‐field cooling and field cooling modes were measured under a 500 Oe magnetic field within the temperature range of 5–340 K on a physical properties measurement system (PPMS, Quantum Design, USA) with the vibrating sample magnetometer option.

### OER Tests

To prepare the working electrodes, 0.75 mL deionized water, 0.25 mL isopropanol, 2 mg carbon powder, 10 mg of electrocatalyst, and 0.1 mL of Nafion 117 solution were mixed under ultrasound for 1 h to form a homogenously mixed ink. Then 3 µL of the ink was dropwise casted onto a specular glassy carbon electrode (≈3 mm in diameter) with the final mass loading of 0.38 mg cm^–2^. OER performances were tested on an electrochemical workstation (Ivium‐n‐Stat, Ivium Technologies) with a standard three‐electrode electrochemical cell. A saturated Ag/AgCl was selected as the reference electrode and a platinum plate as the counter electrode. All LSV curves (and Tafel plots) were measured in the 1 m NaOH aqueous solution with a scan rate of 5 mV s^–1^. The applied potentials were *iR*‐corrected manually and converted to be versus RHE according to the Nernst Equation, where *i* is referred to the current during the LSV tests and *R* is the solution resistance obtained from the Nyquist plots, which were recorded from electrochemical impedance spectroscopy tests at 1.6 V versus RHE over frequencies of 1–10^5^ Hz.

## Conflict of Interest

The authors declare no conflict of interest.

## Supporting information

Supporting Information

## Data Availability

Data available on request from the authors.
