# Supplementary material for: Revealing the Correlation of OER with Magnetism: A New Descriptor of Curie/Neel Temperature for Magnetic Electrocatalysts
Source: Adv Sci (Weinh). 2021 Jul 5;8(17):2101000. doi: 10.1002/advs.202101000 (PMC8425880; doi:10.1002/advs.202101000)
Supplement: Supplementary file 1 — Supporting Information [file ADVS-8-2101000-s001.pdf]

WILEY-VCH

© 2021 Wiley-VCH GmbH

ADVANCED SCIENCE
Open Access

Supporting Information

for *Adv. Sci.*, DOI: 10.1002/advs.202101000

Revealing the correlation of OER with magnetism: A new descriptor of Curie/Neel temperature for magnetic electrocatalysts

Xiaoning Li, Ying Bai, and Zhenxiang Cheng**

© 2021 Wiley-VCH GmbH

Supporting Information

Revealing the correlation of OER with magnetism: A new descriptor of Curie/Neel temperature for magnetic electrocatalysts

Xiaoning Li, Ying Bai, and Zhenxiang Cheng**

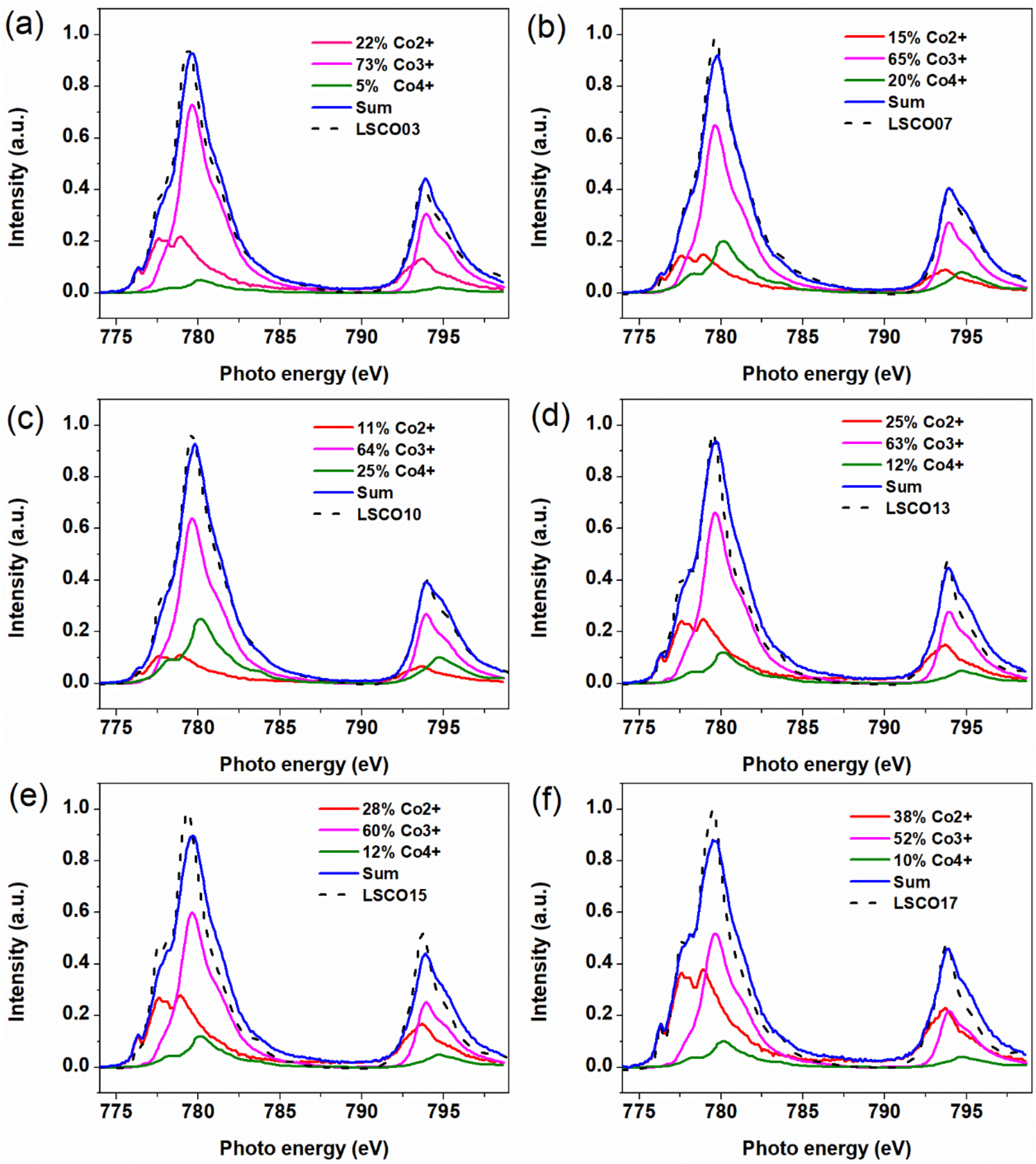

Figure S1. Fitting of Co *L*-edge XANES spectra for all the samples with different $Co^{2+}/Co^{3+}/Co^{4+}$ ratios.

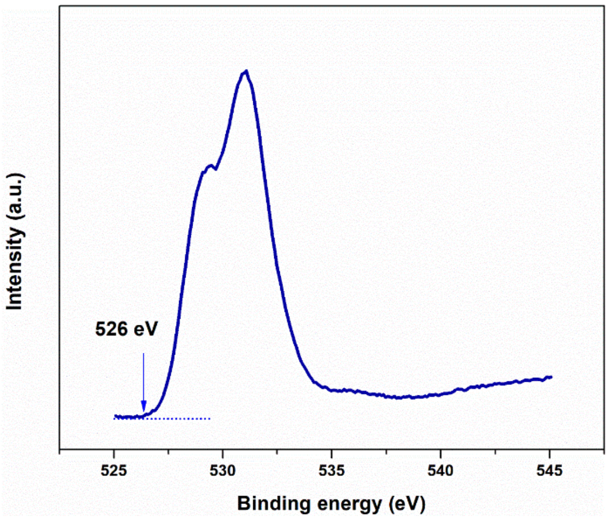

Figure S2. XAS spectrum of O 1s core-level on the sample of LSCO10.

Table S1. Lattice parameters refined by Retiveld method

	LSCO03	LSCO07		LSCO10	LSCO13
Phases	*R-3c*	*R-3c*(77%)	*Pm-3m*(23%)	*Pm-3m*	*Pm-3m*
a	5.449	5.412	3.807	3.833	3.846
b	5.449	5.412	3.807	3.833	3.846
c	13.16	13.08	3.807	3.833	3.846
α	90.00	90.00	90.00	90.00	90.00
β	90.00	90.00	90.00	90.00	90.00
γ	120.0	120.0	90.00	90.00	90.00
Volume	338.3	332.0	55.18	56.33	56.91
∠(Co-O-Co)	172.8	166.7	180.0	180.0	180.0
d(Co-O)	1.921	1.918	1.903	1.917	1.923

Magnetic properties and useful parameters for OER

Figure S3a-f are the temperature dependent magnetization (*M*-*T*) curves measured in the zero-field cooling (ZFC) and field cooling (FC) modes. The main difference between the two modes lies in whether a magnetic field is applied during the cooling process, wherein a magnetic field of 500 Oe is applied during cooling before data is collected in the warming process for the latter mode. The ZFC and FC curves of all samples are coinciding with each other before diverging at certain low temperatures. This originates in the fact that when long-range (ferro/ferri/antiferro-) magnetic orderings exist, the magnetic moments will be aligned along the applied magnetic field, which will be frozen when cooled down below the diverging point. In this respect, larger magnetization is recorded in the FC curve below the diverging

point. However, this kind of discrepancy is vanished given the thermal fluctuation is high enough to disorder any magnetic moments. The Curie/Neel temperature ($T_{C/N}$) is a temperature point when all long-range ordered magnetic moments are thermally disordered, above which materials will be paramagnetic[1, 2]. Hence, the first lowest point in the differentiation of dM/dT based on FC curves can be regarded as $T_{C/N}$, as marked in Figure S3 with arrows.

There are two transition temperatures in the *M*-*T* curve of LSCO07 sample, which may be explained by the fact that it contains 23% perovskite in *Pm*-*3m* apart from the majority in *R*-*3c*. The lower one around 75 K may be the transition of the magnetic states concerning the electron exchange interactions in the *R*-*3c* perovskite, similar to the situation in the LSCO03. The other one around 230 K may be associated with the electron exchange interactions in the *Pm*-*3m* perovskite, similar to the situation of LSCO10. As the active sites of these two phases contribute to the OER differently, and OER performance of the mixture is the weighted average collection, thus it is more reasonable and accurately to use the weighted average temperature as the $T_{C/N}$ instead to reflect the true rule. Thus, the $T_{C/N}$ value is 39 K, 111 K, 248 K, 181 K, 158 K, and 42 K for LSCO03, LSCO07, LSCO10, LSCO13, LSCO15, and LSCO17, respectively.

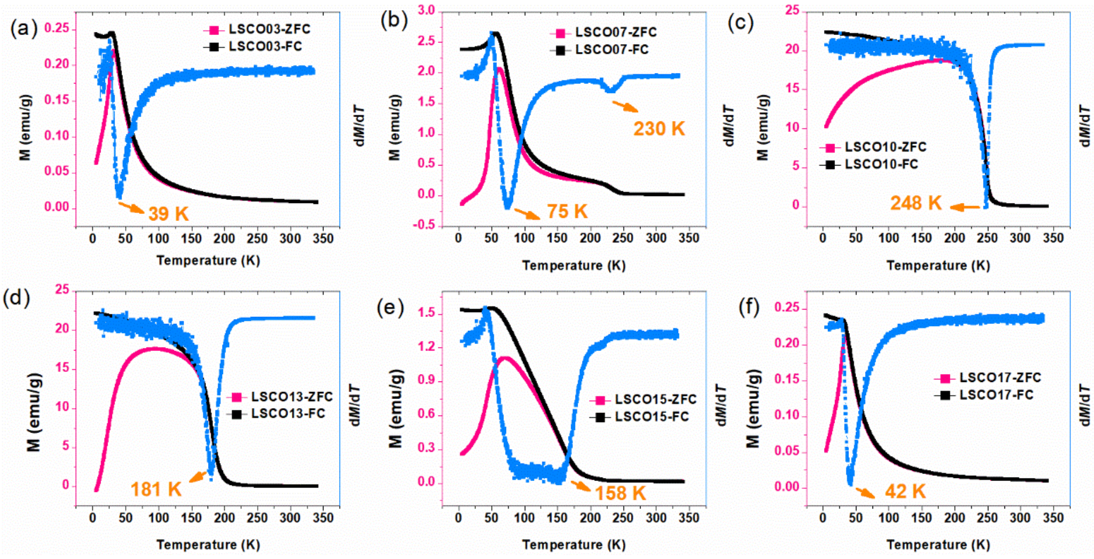

Figure S3. a-f) *M-T* curves measured in ZFC and FC modes under a 500 Oe magnetic field for LSCO03, LSCO07, LSCO10, LSCO13, LSCO15 and LSCO17, respectively.

The plots of reciprocal magnetic susceptibility against temperature ($1/\chi$-T) obtained from FC curves are displayed in **Figure S4**a. Curie–Weiss law can be applied to fit the linear paramagnetic region above the $T_{C/N}$ of FC curves with the **Equation S1**[3].

$$\frac{1}{\chi} = \frac{T}{C} - \frac{T_C}{C} \qquad \text{(S1)}$$

The Curie constant C equals to the reciprocal of the slope of resultant fitting lines, while the intersection on the T-axis is regarded as paramagnetic Curie-Weiss temperature or Weiss temperature (T_θ)[4]. Different from the Curie/Neel temperature ($T_{C/N}$), the Weiss temperature (T_θ) is essentially the arithmetic average of the interspin coupling constants (exchange integrals) of the Hamiltonian[5]. When ferromagnetic exchange coupling overwhelms antiferromagnetic one, T_θ will be positive. On the contrary, a negative T_θ is obtained instead. For the paramagnetic materials without a T_C, T_θ should be near zero when fitting the whole region with Curie–Weiss law in theory. Furthermore, the effective magnetic moment (U_{eff}) and possible spin configuration can be roughly estimated based on the Curie constant C from Curie-Weiss fitting of paramagnetic region, according to the **Equation S2, 3**[3]

$$U_{eff} = \sqrt{\frac{3kC}{N}} \qquad (2)$$

$$U_{eff} = g\sqrt{J(J+1)}\,\mu_B \qquad (3)$$

where k and N are the Boltzmann constant and the number of magnetic ions in the unit cell respectively, g is the Lande factor, J is the total quantum number and μ_B is the Bohr magneton.

The Lande factor g is 2.0023 for a free electron, and for the condense matters it can be estimated by electron spin resonance (ESR) measurements. With g known, the spin quantum number S can be calculated, as it is equaled to J when the total quantum number L=0 in the 3d

transition metal oxides. Then, the specific spin configuration and e_g electron number can be estimated with the consideration that the number of unpaired electron is $2\times S$ [3]. Here, the e_g electron number is not the main subject in present work, so we just plot the resultant T_θ, U_{eff} and $T_{C/N}$ obtained from magnetic measurements (Figure S4b). Remarkably, $T_{C/N}$ is increasing at first and decreasing afterwards with increasing of Sr content, achieving the maximum in the LSCO10. However, when it comes to T_θ and U_{eff}, a negative T_θ is observed at LSCO15 and LSCO17 together with a jump of U_{eff}. The distinctiveness of T_θ and U_{eff} from $T_{C/N}$ is reckonable, as $T_{C/N}$ is proportional to the orbital hybridization while T_θ is indicative for antiferro-/ferro- magnetic ground states and U_{eff} is indicative for unpaired electron numbers.

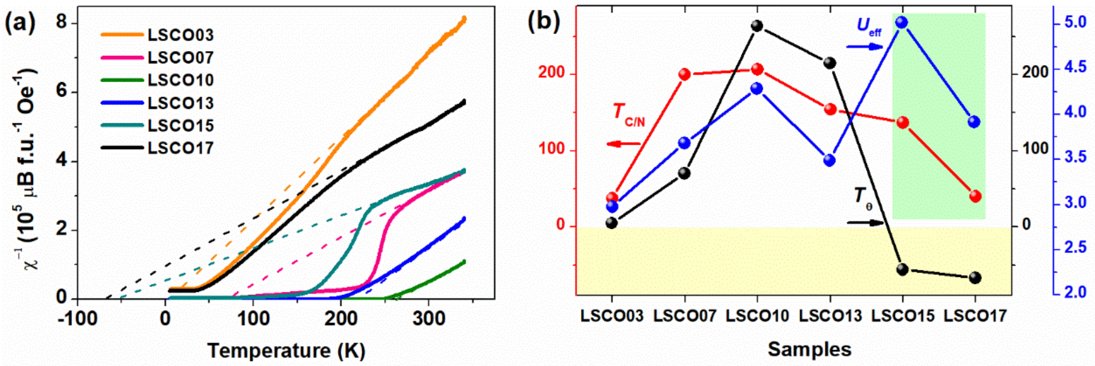

Figure S4. a) $1/\chi$-T curves based on the FC data; b) evolution of magnetic parameters $T_{C/N}$, T_θ and U_{eff}.

References

[1] D. R. Behrendt, S. Legvold, F. H. Spedding, *Phys. Rev.* **1958**, *109*, 1544.

[2] M. Tadic, D. Nikolic, M. Panjan, G. R. Blake, *J. Alloy Compd.* **2015**, *647*, 1061.

[3] X. Li, Y. Sun, Q. Wu, H. Liu, W. Gu, X. Wang, Z. Cheng, Z. Fu, Y. Lu, *J. Am. Chem. Soc.* **2019**, *141*, 3121.

[4] H. Taguchi, M. Shimada, M. Koizumi, *Mat. Res. Bull.* **1978**, *13*, 1225.

[5] A. Czachor, *J. Magn. Magn. Mater.* **1995**, *139*, 355.
